# A Clinical Commentary on Facial Botulinum Toxin A Use Among Transgender Women: Unique Considerations Among Gender‐Diverse Populations

**DOI:** 10.1111/jocd.70774

**Published:** 2026-04-02

**Authors:** Vincent Pecora, Rayva Khanna, Pooja Sodha

**Affiliations:** ^1^ George Washington University School of Medicine and Health Sciences Washington DC USA; ^2^ Department of Dermatology MedStar Washington Hospital Center/Georgetown University Washington DC USA

## 1 Introduction

Non‐invasive dermatological procedures such as neuromodulators and injectable filler are often sought out in LGBTQ+ and gender‐diverse patient populations [1]. While these procedures are often depicted as being sought out for purely cosmetic or aesthetic purposes, dermatological care among gender‐diverse patients is influenced by psychosocial factors such as gender dysphoria and the pursuit of gender‐affirming care [1, 2]. Facial feminization surgery (FFS) is highly effective in relieving gender dysphoria and recontouring facial structures. However, these procedures are expensive, time‐consuming, and have higher rates of post‐intervention complications. Non‐surgical techniques such as botulinum toxin A are common interventions that aid in recontouring facial structures for the purpose of gender‐affirmation among transgender women and are less expensive compared to FFS procedures [2, 3, 4]. Still, there are currently no studies summarizing the use of facial botulinum toxin A among transgender women. Thus, this review sought to identify unique considerations for facial botulinum toxin A use among transgender women.

## 2 Materials and Methods

From November 1, 2025, to January 21, 2026, a literature search was conducted to evaluate the use of botulinum toxin A among transgender women. The search terms “transgender,” “transfeminine,” “gender diverse,” “trans woman,” “non‐surgical,” “botulinum toxin,” “botulinum toxin A,” and “Botox” were used in PubMed, Google Scholar, and Scopus. Studies were excluded if they did not include transgender women, discussed surgical feminization techniques, focused on areas aside from the face, or did not explicitly mention the use of botulinum toxin A. Only studies with measurable outcomes and studies that did not include expert opinions, procedural descriptions, or ethical analyses were selected for inclusion. Study types including case reports, case series, cohort studies, case–control, retrospective, prospective studies, and cross‐sectional studies were included in search criteria.

## 3 Results

A total of 15 studies discussed facial botulinum toxin A (BoNT‐A) use among transgender women. From our literature search, the use of BoNT‐A in gender‐affirming care, anatomical considerations for BoNT‐A, injection techniques, estrogen considerations, complications of injections, and barriers to care were extracted for analysis and discussion.

## 4 Discussion

### 4.1 Anatomical Considerations

In the upper face, botulinum toxin A is commonly used to raise the lateral brow, soften forehead wrinkles and brow ridges, widen eye aperture, and decrease sebaceous activity and pore appearance [5, 6]. Botulinum toxin A was also shown to be effective in the reduction of rhytid appearance, especially when used in combination with injectable filler for volume restoration [7]. Injection into the supraorbital region of the orbicularis oculi muscle was also effective in raising the lateral brow which was highly desired among transgender women [8]. Injection of botulinum toxin A extending past the lateral brow border may minimize brow arch appearance. Thus, it is important for dermatologists to directly inject into glabellar complex muscles including the procerus, corrugator muscles, and superolateral orbicularis oculi without extension beyond the brow peak [9]. Transgender women have also been shown to seek out larger eyes and a widened interocular aperture. Flynn and colleagues demonstrated how injecting Botox to the crow's feet and pretarsal inferior orbicularis oculi muscle led to a larger eye appearance and achieved facial feminization goals among a case series of transgender women [10]. Direct injection into the palpebral surface of the orbicularis oculi muscle has also been shown to be effective in widening the eye aperture, with injections primarily placed along the lower eyelid [11].

In the middle face region, BoNT‐A was used to reduce sebaceous activity in malar surfaces, lift the nasal tip, and augment the zygomatic arches [6]. Injection into the depressor septi nasi muscle was effective in reducing nasal alar flare and creating a lifted appearance of the nasal tip [8]. Inducing atrophy of the glabellar musculature has been shown to create a more obtuse nasofrontal angle and a more feminized nasal structure [12].

For the lower facial regions, BoNT‐A was used to induce atrophy of the masseter muscles, achieve a slimmer facial architecture, and augment chin shape [6, 7]. Direct injection of BoNT‐A into the masseter muscles helped minimize prominence of the jawline and created a “heart‐shaped” facial architecture that was highly sought out by several transgender patients [8]. A recent study demonstrated that injection of botulinum toxin in the lower face for transversal masseter reduction among a cohort of transgender women led to significant improvements in satisfaction with lower face contouring outcomes [13].

### 4.2 Injection Approaches

Transgender women used an average of 74 units of incobotulinumtoxinA in the upper face, microdroplets with 0.5 units per injection on each side of the middle face, and 43 units of incobotulinumtoxinA in the lower facial regions [6]. Many transgender women reported seeking out lifted eyebrow arches as components of their dermatological gender‐affirming care [9]. Patients received a range between 4 and 12 units of onabotulinumtoxinA on the lateral aspect of supraorbital ridges in order to create a lifted eyebrow architecture [6, 8]. Many patients also reported seeking out slimmer facial architecture through inducing masseter muscle atrophy [9]. Treatment of masseter hypertrophy required greater units, ranging between 20 and 35 units of incobotulinumtoxinA on each side of the face [6]. Given the widespread use of BoNT‐A to achieve facial feminization, it is important for dermatologists to understand the quantity, quality, and approaches of injections.

### 4.3 Complications and Barriers

Common complications of botulinum toxin A include pain at injection sites, swelling, asymmetry, ptosis, and redness. Rarely, botulinum toxin A may cause local infections, abscesses, or paresthesias [14]. While complications for botulinum toxin A use are rare, when used in conjunction with injectable filler, the risk of adverse effects including filler migration, skin irritation, and ptosis increases [9]. Since many transgender women utilize both botulinum toxin A and filler in their pursuit of gender‐affirming care, it is crucial for dermatologists to understand and monitor for these effects.

Many transgender women utilize exogenous estrogen therapy as components of their gender‐affirming care [9, 10]. While there are scattered animal studies demonstrating possible correlations between botulinum toxin A administration and hormonal dysregulations, these findings are largely limited given the absence of clear research in human trials. Exogenous estrogen therapy is frequently depicted as drastically increasing the risk of developing DVTs and polycythemia. However, no studies to date have shown macro‐ or microvascular occlusions secondary to direct botulinum toxin A injection in the face among transgender women on hormonal therapy.

Barriers to access for botulinum toxin A among transgender women influence the pursuit of these procedures. The most common barriers include cost concerns [9]. Over half of transgender women pursuing botulinum toxin A reported experiencing housing insecurity and many individuals earned less than minimum wage [9]. This led to increased rates of seeking care from non‐healthcare professionals using non‐FDA approved injectables including silicone for facial and body contouring [9].

Distrust in the medical system also impacts the pursuit of care among gender‐diverse patients [15]. Many LGBTQ+ individuals report feeling misunderstood or discriminated against by medical professionals [15, 16, 17]. It is therefore important for providers to implement strategies that increase comfort and satisfaction among gender‐diverse patients. Physicians are encouraged to inquire about preferred names and pronouns, learn about appropriate terminology, and display statements of non‐discrimination in clinic settings [15, 18]. Training about LGBTQ+ dermatology topics is also poor, which has led to gaps in knowledge among dermatologists [19, 20]. In order to adequately understand and manage gender‐diverse patients, it is crucial to incorporate education about LBGTQ+ dermatology into standardized dermatology curriculum.

## 5 Limitations

The majority of research regarding aesthetic considerations, injection techniques, and adverse effects of facial BoNT‐A occurs in cisgender women. The present study is limited by the relatively small sample population, which is indicative of the lack of research for healthcare outcomes among gender‐diverse populations.

## 6 Conclusion

Botulinum toxin A offers a unique non‐invasive opportunity to achieve facial feminization among transgender women. Facial feminization interventions among transgender women have been shown to reduce rates of gender dysphoria [3]. As components of gender‐affirming care, many patients seek out non‐invasive procedures including botulinum toxin A and injectable filler before pursuing more invasive and expensive surgical options [2]. The pursuit of these procedures can significantly impact social functioning and improve mental health among both transgender men and transgender women. Anatomical considerations and desired aesthetic outcomes by facial region are crucial to understand when administering botulinum toxin A injections [20, 21]. Barriers to access among transgender women are common, which may lead to possibly dangerous pursuits of dermatological procedures from unlicensed practitioners. Future directions for non‐invasive facial feminization include neck contouring and Nefertiti facelifts, although these procedures require more research [22]. Overall, dermatologists must understand the unique considerations of botulinum toxin A use among transgender women to provide comprehensive, inclusive care to underrepresented LGBTQ+ patient populations.

## Author Contributions

V.P. was involved in project conceptualization, literature search, data analysis, manuscript writing, and submission. R.K. and P.S. were responsible for project conceptualization and manuscript writing.

## Funding

The authors have nothing to report.

## Ethics Statement

The authors confirm that the ethical policies of the journal, as noted on the journal's author guidelines page, have been adhered to.

## Conflicts of Interest

The authors declare no conflicts of interest.

## Data Availability

The data that support the findings of this study are available from the corresponding author upon reasonable request.

## References

[1] B. A. Ginsberg , M. Calderon , N. M. Seminara , and D. Day , “A Potential Role for the Dermatologist in the Physical Transformation of Transgender People: A Survey of Attitudes and Practices Within the Transgender Community,” Journal of the American Academy of Dermatology 74, no. 2 (2016): 303–308, 10.1016/j.jaad.2015.10.013. 26669479

[2] S. D. Morrison , K. S. Vyas , S. Motakef , et al., “Facial Feminization: Systematic Review of the Literature,” Plastic and Reconstructive Surgery 137, no. 6 (2016): 1759–1770, 10.1097/PRS.0000000000002171. 27219232

[3] D. Coon , J. Berli , N. Oles , et al., “Facial Gender Surgery: Systematic Review and Evidence‐Based Consensus Guidelines From the International Facial Gender Symposium,” Plastic and Reconstructive Surgery 1 (2022): 212–224, 10.1097/PRS.0000000000008668. 34936625

[4] M. Zaliznyak , C. Bresee , and M. M. Garcia , “Age at First Experience of Gender Dysphoria Among Transgender Adults Seeking Gender‐Affirming Surgery,” JAMA Network Open 3, no. 3 (2020): e201236, 10.1001/jamanetworkopen.2020.1236. 32176303 PMC7076333

[5] D. M. Tordoff , J. W. Wanta , A. Collin , C. Stepney , D. J. Inwards‐Breland , and K. Ahrens , “Mental Health Outcomes in Transgender and Nonbinary Youths Receiving Gender‐Affirming Care,” JAMA Network Open 5, no. 2 (2022): e220978, 10.1001/jamanetworkopen.2022.0978. 35212746 PMC8881768

[6] B. Viscomi , “From Anatomical Modifications to Skin Quality: Case Series of Botulinum Toxin and Facial Fillers for Facial Feminization in Transgender Women,” Clinical, Cosmetic and Investigational Dermatology 15 (2022): 1333–1345, 10.2147/CCID.S363882. 35860607 PMC9293247

[7] M. Ascha , M. A. Swanson , J. P. Massie , et al., “Nonsurgical Management of Facial Masculinization and Feminization,” Aesthetic Surgery Journal 39, no. 5 (2019): NP123–NP137, 10.1093/asj/sjy253. 30383180

[8] K. De Boulle , N. Furuyama , I. Heydenrych , et al., “Considerations for the Use of Minimally Invasive Aesthetic Procedures for Facial Remodeling in Transgender Individuals,” Clinical, Cosmetic and Investigational Dermatology 14 (2021): 513–525, 10.2147/CCID.S304032. 34012284 PMC8128506

[9] P. H. Rambhia , T. Keaney , Y. C. Chang , A. Chapas , and J. MacGregor , “Aesthetic Considerations for Neuromodulator Use in Transgender Patients,” Dermatologic Surgery 50, no. 9S (2024): S80–S84, 10.1097/DSS.0000000000004325. 39196839

[10] T. C. Flynn , J. A. Carruthers , and J. A. Carruthers , “Botulinum‐A Toxin Treatment of the Lower Eyelid Improves Infraorbital Rhytides and Widens the Eye,” Dermatologic Surgery 27 (2001): 703–708. 11493292 10.1046/j.1524-4725.2001.01038.x

[11] M. de Maio , A. Swift , M. Signorini , and S. Fagien , “Facial Assessment and Injection Guide for Botulinum Toxin and Injectable Hyaluronic Acid Fillers: Focus on the Upper Face,” Plastic and Reconstructive Surgery 140, no. 2 (2017): 265e–276e. 10.1097/PRS.0000000000003544 28746271

[12] N. Dhingra , L. M. Bonati , E. B. Wang , M. Chou , and J. Jagdeo , “Medical and Aesthetic Procedural Dermatology Recommendations for Transgender Patients Undergoing Transition,” Journal of the American Academy of Dermatology 80 (2019): 1712–1721. 30678999 10.1016/j.jaad.2018.05.1259

[13] M. Slimani , E. Ramelli , L. Azoulai , M. Atlan , A. G. Lellouch , and S. Cristofari , “Transwomen Satisfaction After Nonsurgical Management of Large Lower Face With Botulinum Toxin Injection of the Masseter: A Preliminary FACE‐Q Study,” Annales de Chirurgie Plastique et Esthétique 71, no. 2 (2025): 186–190, 10.1016/j.anplas.2025.09.003. 41027806

[14] H. Witmanowski and K. Błochowiak , “The Whole Truth About Botulinum Toxin—A Review,” Postepy Dermatologii I Alergologii 37, no. 6 (2020): 853–861, 10.5114/ada.2019.82795. 33603602 PMC7874868

[15] N. Bhatt , J. Cannella , and J. P. Gentile , “Gender‐Affirming Care for Transgender Patients,” Innovations in Clinical Neuroscience 19, no. 4–6 (2022): 23–32. 35958971 PMC9341318

[16] A. Montero , L. Hamel , S. Artiga , and L. Dawson , “LGBT Adults' Experiences With Discrimination and Health Care Disparities: Findings From the KFF Survey of Racism, Discrimination, and Health,” KFF (2024), https://www.kff.org/racial‐equity‐and‐health‐policy/poll‐finding/lgbt‐adults‐experiences‐with‐discrimination‐and‐health‐care‐disparities‐findings‐from‐the‐kff‐survey‐of‐racism‐discrimination‐and‐health/#:~:text=LGBT%20adults%20who%20have%20used,in%20the%20past%20three%20years.

[17] N. M. Lampe , H. Barbee , N. M. Tran , S. Bastow , and T. McKay , “Health Disparities Among Lesbian, Gay, Bisexual, Transgender, and Queer Older Adults: A Structural Competency Approach,” International Journal of Aging & Human Development 98, no. 1 (2024): 39–55, 10.1177/00914150231171838. 37122150 PMC10598237

[18] L. Shields , T. Stovall , and H. Colby , “Increasing Inclusivity and Reducing Reactance During Provider‐Patient Interactions,” Medical Decision Making 43, no. 4 (2023): 478–486, 10.1177/0272989X231156430. 36825755

[19] J. T. Hyde , J. C. Trinidad , K. T. Shahwan , C. Nguyen , H. Yeung , and D. R. Carr , “Learning Experiences in LGBT Health During Dermatology Residency,” Cutis 110, no. 4 (2022): 215–219, 10.12788/cutis.0626. 36446104

[20] M. S. Gart and K. A. Gutowski , “Overview of Botulinum Toxins for Aesthetic Uses,” Clinics in Plastic Surgery 43, no. 3 (2016): 459–471, 10.1016/j.cps.2016.03.003. 27363760

[21] S. Elhachimi , D. Z. Liao , M. Gray , and J. Rosenberg , “Nonsurgical Interventions for Gender‐Affirming Facial Feminization: A Scoping Review,” Facial Plastic Surgery & Aesthetic Medicine 27, no. 4 (2025): 361–368, 10.1089/fpsam.2024.0091. 39504134

[22] C. J. Salgado , A. G. Nugent , T. Satterwaite , K. H. Carruthers , and N. R. Joumblat , “Gender Reassignment: Feminization and Masculinization of the Neck,” Clinics in Plastic Surgery 45, no. 4 (2018): 635–645, 10.1016/j.cps.2018.06.006. 30268248

